# The Evolution of Dental Journals from 2003 to 2012: A Bibliometric Analysis

**DOI:** 10.1371/journal.pone.0119503

**Published:** 2015-03-17

**Authors:** Yasas Shri Nalaka Jayaratne, Roger Arthur Zwahlen

**Affiliations:** 1 Division of Orthodontics, Department of Craniofacial Sciences, University of Connecticut School of Dental Medicine, 263 Farmington Avenue, Farmington, Connecticut 06030, United States of America; 2 Discipline of Oral & Maxillofacial Surgery, Faculty of Dentistry, The University of Hong Kong, Pokfulam, Hong Kong Special Administrative Region; Mathematical Institute, HUNGARY

## Abstract

Bibliometrics are a set of methods, which can be used to analyze academic literature quantitatively and its changes over time. The objectives of this study were 1) to evaluate trends related to academic performance of dental journals from 2003 to 2012 using bibliometric indices, and 2) monitor the changes of the five dental journals with the highest and lowest impact factor (IF) published in 2003. Data for the subject category "Dentistry, Oral Surgery & Medicine" was retrieved from the Journal Citation Reports (JCR) published from 2003 to 2012. Linear regressions analysis was used to determine statistical trends over the years with each bibliometric indicator as the dependent variable and the JCR year as the predictor variable. Statistically significant rise in the total number of dental journals, the number of all articles with the steepest rise observed for research articles, the number of citations and the aggregate IF was observed from 2003 to 2012. The analysis of the five top and five bottom-tire dental journals revealed a rise in IF however, with a wide variation in relation to the magnitude of this rise. Although the IF of the top five journals remained relatively constant, the percentile ranks of the four lowest ranking journals in 2003 increased significantly with the sharpest rise being noted for the British Journal of Oral & Maxillofacial Surgery. This study revealed significant growth of dental literature in absolute terms, as well as upward trends for most of the citation-based bibliometric indices from 2003 to 2012.

## Introduction

Journals play a vital role in academia by disseminating scholarly and technical work, evaluating and peer-review of research, archiving of such work and serving as basis for scholarly credits [1]. Peer-reviewed publications have been designated as the “primary mode of communication and record for scientific research” [2]. Olk and Griffith in 2004 argued that journals still represent the primary source of knowledge in a given field. They stated that scholars push the boundaries of their field but journals are needed to advance the main body of knowledge [3]. The world’s first dental journal, *The American Journal of Dental Science*, started its publication in 1839 [4]. Since then the journals in the field of dentistry have been acting as a source of knowledge and mode of communication within the dental community and other disciplines.

Many changes can occur in the lifetime of a single academic journal or a in a group of journals. Thus, reliable techniques to document and analyze such changes are needed. “Bibliometrics” are a set of methods to analyze academic literature quantitatively [5]. Basically this term means “the application of quantitative analysis and statistics to publications such as journal articles and their accompanying citation counts”[6]. Such analyses can be used to evaluate the impact of publications at different echelons including the level of a single author, a group of researchers, a specific paper, a journal, a particular field, or an academic institution.

The impact factor (IF) published by Thomson Reuters’ annual Journal Citation Reports (JCR) remains to be a popular bibliometric index. The journal IF indicates the average number of times articles published two years ago were cited in the JCR year. It is calculated by dividing the total number of citations to a particular journal in the JCR year by the number of articles published in that journal in the past two years [7]. The JCR contains a number of bibliometric data and indices other than the IF, which can reveal important information about the performance of each journal. Moreover, it is possible to retrieve from JCR, aggregate data for all journals belonging to a certain subject category. Such “category data” enables the evaluation of bibliometric trends within a particular specialty as a whole.

The progress of dental journals has not been studied in the past. With bibliometric information, it is possible to gauge the growth and other characteristic changes in the field. Consequently, insights can be gained on how dentistry’s collective body of knowledge is evolving over time. Therefore, this study was designated 1) to evaluate trends related to academic performance of dental journals from 2003 to 2012 using bibliometric indices, and 2) to monitor changes of the five dental journals with the highest and lowest IF’s published in 2003 and remaining in circulation up to 2012.

## Methods

### Data source

The science edition of the Journal Citation Reports (JCR) available on the ISI Web of Knowledge database was used for this study. Data available for the subject category grouped in the JCR as “Dentistry, Oral Surgery & Medicine” was searched from 2003 to 2012. Year 2003 was used as the starting point because subject category data were introduced from this year onwards, while 2012 was used as the endpoint, as the latest available JCR was of this year. The raw data is available from the Journal Citation Reports published by Thomson Reuters. However, users need to have a subscription to access this database.

### Bibliometric indicators

Several bibliometric indicators were collected from the subject category page for ‘Dentistry, Oral Surgery & Medicine’ for each JCR year. Descriptions of these indicators, as presented by its publisher [7] are shown below in italics:

1. Number of journals
2. Their publication frequency (annual, semiannual, quarterly, bimonthly, monthly)
3. Total cites—*the total number of citations to journals in the subject category in the JCR year*
4. Median impact factor—*the median value of all journal impact factors in the subject category*
5. Aggregate impact factor—*calculated the same way as the Impact Factor for a journal*, *but it takes into account the number of citations to all journals in the category and the number of articles from all journals in the category*
6. Aggregate immediacy index—*The Immediacy Index is the average number of times an article is cited in the year it is published*. *The Immediacy Index is calculated by dividing the number of citations to articles published in a given year by the number of articles published in that year*. *The aggregate Immediacy Index indicates how quickly articles in a subject category are cited*.
7. Aggregate cited half-life—*the median age of the articles that were cited in the JCR year*. *The aggregate cited half-life is an indication of the turnover rate of the body of work on a subject*.
8. Aggregate citing half-life—*the median age of articles cited by journal in the category in the JCR year*
9. Number of research articles, reviews and other publication types (e.g. editorials, letters etc.) published in the JCR year
10. Ratio between number of references and publication count for each of the aforementioned article types

### Longitudinal changes of those five dental journals with the highest and lowest IF’s published in 2003

The summary list of 2003 JCR arranged according to the IF was used to identify 10 journals with the highest and lowest ranks (i.e. five journals from each end of the spectrum). The IF, rank, total cites, immediacy index, number of citable publications (articles and reviews), cited half-life and citing half-life of each of these 10 journals from 2003 to 2012 were retrieved from the JCR of the respective year. Only journals that continued their publications until 2012 were considered. The journal ‘Critical Reviews in Oral Biology and Medicine’ was excluded from this analysis as it ceased publication as an independent journal in 2004 and merged subsequently with the Journal of Dental Research. Since the number of journals with an IF differed from year to year, we computed the percentile rank of each journal instead of using the raw rank available from the JCR.

### Data analysis

Linear regressions were used to determine statistical trends over the years. Following López-Abente and Muñoz-Tinoco [8] each bibliometric indicator was used as the dependent variable while the JCR year was used as the predictor variable. The same method has been used in other clinical disciplines for analyzing bibliometric data trends [9–14]. The slope of the regression (β), its significance and the coefficient of determination (R^2^) were recorded. The β value indicates the average annual change of a given bibliometric indicator, while the R^2^ value denotes how well the regression line approximates the actual data points. For example, R^2^ = 1 would mean that the regression line perfectly fits the data. The null hypothesis of this model is that β = 0. If the p-value is less than 0.05, the null hypothesis is rejected. Hence it can be concluded that there is a significant relationship between the variables in the linear regression model.

The Pearson correlation coefficient (r) between combined research articles and reviews count, total cites, median IF, aggregate IF and aggregate immediacy index was calculated. The combined research articles and reviews count was used as these are the only publication types that are used when computing the IF. All statistical tests were performed with IBM SPSS 20 software (IBM, New York, USA).

## Results

### Changes related to all dental journals

**Fig 1:**
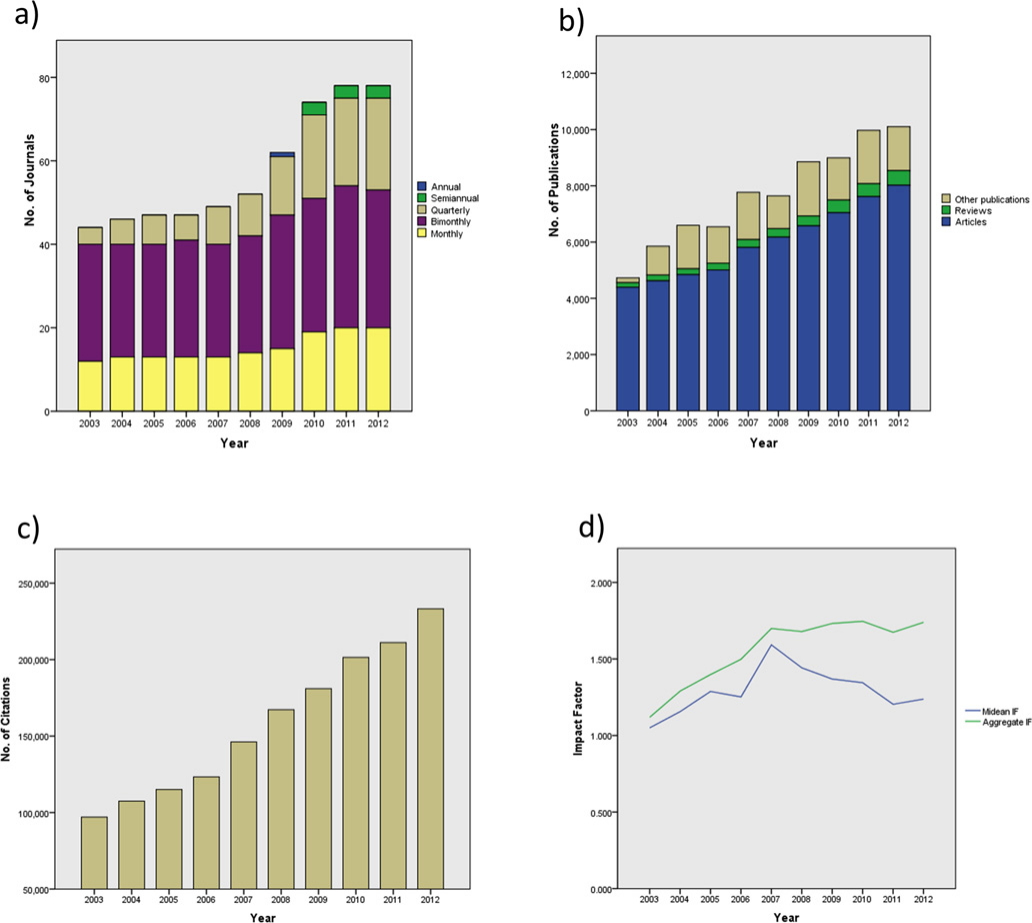
Changes in key publication metrics of all dental journals from 2003 to 2012. a) number of journals b) number of publications c) number of citations and d) median IF and aggregate IF.

The total number of dental journals increased significantly from 46 in 2003 to 83 in 2012 (β = 4.564, p< 0.001, R^2^ = 0.876). Although an increase in bimonthly and monthly journals was noted (Fig. 1 and Table 1), the most sharp rise was observed in quarterly journals (β = 2.164, p< 0.001, R^2^ = 0.931). The number of citations received by dental journals more than doubled from 97,081 in 2003 to 233,232 in 2012. Regression analysis revealed significant rise of citation counts throughout the years (β = 15,616, p<0.001, R^2^ = 0.985). The changes in the median IF of dental journals was not statistically significant (p = 0.397) but a significant rise in the aggregate IF was seen (β = 0.065, p = 0.001, R^2^ = 0.787). The aggregate immediacy Index also demonstrated a significant rise from 0.159 in 2003 to 0.275 in 2012 (β = 0.013, p < 0.001, R^2^ = 0.833). A statistically significant decline in the aggregate cited half-life was found (β = - 0.06, p = 0.002, R^2^ = 0.726) but the change of the aggregate citing half-life was not significant (p = 0.090).

**Table 1:** Changes in the key publication metrics of dental journals from 2003 to 2012.

Metric		2003	2004	2005	2006	2007	2008	2009	2010	2011	2012	β	p-value	R^2^
**Publication frequency**	Annual	0	0	0	0	0	0	1	0	0	0	0.018	0.631	0.030
	Semiannual	0	0	0	0	0	0	0	3	3	3	0.382	0.006	0.636
	Quarterly	4	6	7	6	9	10	14	20	21	22	2.164	< 0.001	0.913
	Bimonthly	28	27	27	28	27	28	32	32	34	33	0.800	0.001	0.750
	Monthly	12	13	13	13	13	14	15	19	20	20	0.958	< 0.001	0.826
	Total No. of Journals	46	48	49	49	51	55	64	77	81	83	4.564	< 0.001	0.876
**Indices**	Total Cites	97081	107503	115087	123328	146199	167243	181025	201478	211097	233232	15616	< 0.001	0.985
	Median Impact Factor	1.05	1.156	1.288	1.252	1.592	1.442	1.369	1.345	1.204	1.238	0.015	0.397	0.091
	Aggregate Impact Factor	1.119	1.29	1.398	1.498	1.699	1.679	1.732	1.746	1.674	1.739	0.065	0.001	0.787
	Aggregate Immediacy Index	0.159	0.179	0.177	0.215	0.238	0.251	0.282	0.243	0.261	0.275	0.013	< 0.001	0.833
	Aggregate Cited Half-life	8.9	8.7	8.7	8.6	8.5	8.4	8.2	8.3	8.4	8.4	-0.060	0.002	0.726
	Aggregate Citing Half-life	9.0	9.0	9.0	8.8	8.8	8.7	8.5	8.6	8.8	8.9	-0.032	0.090	0.316
**Number of references per publication**	Articles	26.8	26.8	27.6	28.0	28.2	29.3	29.1	29.5	30.6	30.5	0.447	< 0.001	0.963
	Reviews	75.5	100.4	89.3	91.9	100.6	79.0	76.3	72.2	67.8	64.8	-2.899	0.031	0.459
	Other	4.1	3.8	2	2.7	2.2	2.8	2.7	2.6	2.7	3.6	-0.052	0.524	0.053

From 2003 to 2012, the total number of publications in dental journals more than doubled from 4,727 to 10,102 papers. The number of all publication types significantly increased, but the β value indicates the steepest rise was evident in research articles (β = 422.224, p < 0.001, R^2^ = 0.982) followed by other publication types (β = 120.182, p < 0.001, R^2^ = 0.500). Reviews recorded the least growth (β = 39.515, p < 0.001, R^2^ = 0.955). The number of references in each publication type increased significantly (p < 0.001). However, number of references per publication in research articles significantly increased (β = 0.447, p < 0.001, R^2^ = 0.963) throughout the years, while a decline in this ratio was observed in the case of reviews (β = -2.899, p = 0.031, R^2^ = 0.459).

The combined article and review count correlated greatly with the total cites (r = 0.998, p < 0.001), aggregate IF (r = 0.840, p = 0.002) and immediacy index (r = 0.883, p = 0.001) but poorly with median IF (r = 0.240, p = 0.504).

### Changes observed in the five dental journals with the highest and lowest IF’s published in 2003

**Fig 2:**
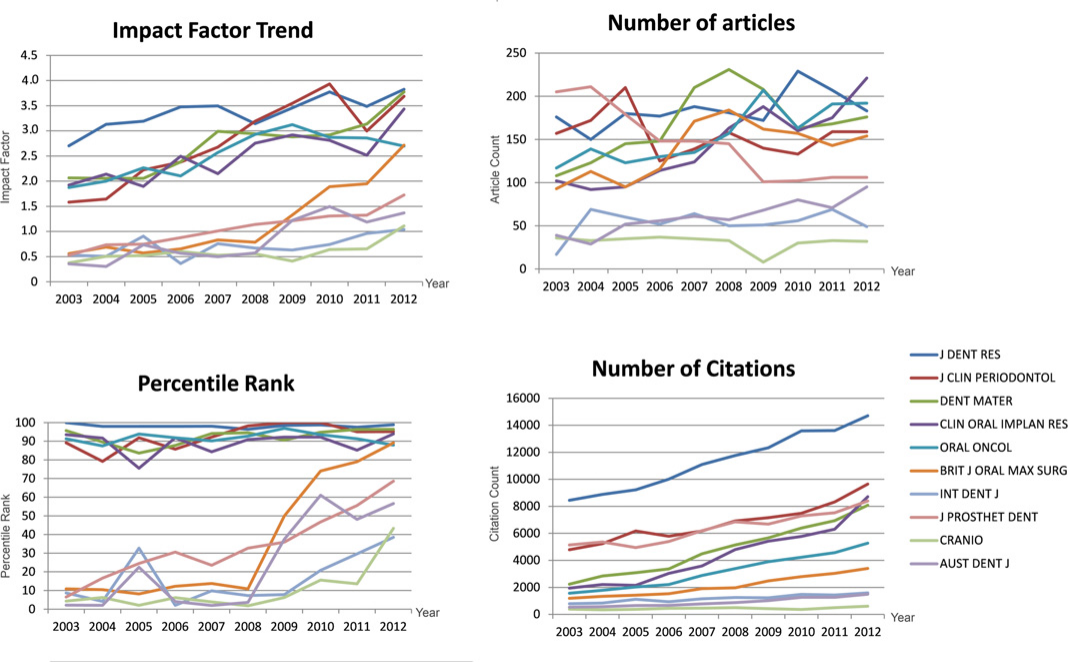
Changes in key publication metrics of individual dental journals from 2003 to 2012

In 2003 the highest-ranking journals in terms of IF were the Journal of Dental Research (IF = 2.702), Journal of Clinical Periodontology (IF = 1.582), Dental Materials (IF = 2.064), Clinical Oral Implants Research (IF = 1.922) and Oral Oncology (IF = 1.876). The five journals with the lowest IF’s included Australian Dental Journal (IF = 0.358), Cranio (IF = 0.375), Journal of Prosthetic Dentistry (IF = 0.527), International Dental Journal (IF = 0.531) and British Journal of Oral & Maxillofacial Surgery (IF = 0.559). For all 10 journals citations increased significantly from 2003 to 2012 (Fig. 2), with the highest year to year rise recorded for Clinical Oral Implants Research (β = 702.87, R^2^ = 0.934, p < 0.001) and the lowest slope noted for Journal of Dental Research (β = 0.001, R^2^ = 0.986, p < 0.001). Similarly, the IF of these journals significantly increased during the same period, but a wide variation was noted in relation to the magnitude of this rise. The Journal of Clinical Periodontology recorded the highest annual IF rise (β = 0.956, R^2^ = 0.114, p < 0.001) whereas the annual IF change in the Journal of Dental Research was the smallest (β = 0.092, R^2^ = 0.687, p = 0.003). Statistically significant changes in relation to the number of articles published were observed only in five journals. The number of articles published in the Journal of Prosthetic Dentistry significantly decreased (β = -13.061, R^2^ = 0.882, p < 0.001) from 2003 to 2012, while those in the Australian Dental Journal, British Journal of Oral & Maxillofacial Surgery, and Oral Oncology increased with Clinical Oral Implants Research journal accounting for the greatest rise (β = 13.564, R^2^ = 0.862, p < 0.001). Out of the five journals with the highest IF’s in 2003, only the rise in the percentile rank of the Journal of Clinical Periodontology was statistically significant (p = 0.022). In contrast, the percentile ranks of four of the lowest ranking journals in 2003 (i.e. British Journal of Oral & Maxillofacial Surgery, International Dental Journal, Journal of Prosthetic Dentistry, Cranio and Australian Dental Journal) increased significantly. The steepest rise was noted for the British Journal of Oral & Maxillofacial Surgery (β = 9.846, R^2^ = 0.794, p < 0.001).

## Discussion

The definition adapted by the JCR for the group of journals under the banner Dentistry, Oral Surgery and Medicine “*covers resources on the anatomy*, *physiology*, *biochemistry*, *and pathology of the teeth and oral cavity*. *This category includes specific resources on periodontal disease*, *dental implants*, *oral and maxillofacial surgery*, *oral pathology*, *and oral surgery*. *Coverage also includes resources on community dentistry*, *public health dentistry*, *and pediatric dentistry*”[15]. Thus, this category of journals covers a wide variety of sub-disciplines ranging from basic sciences to clinical specialties.

As far as the authors are aware, this is the only study that quantitatively evaluated the academic performance of dental journals in the past 10 years. We found significant growth in the dental literature in terms of number of journals, publications and acquired citations. Majority of the key bibliometric indices demonstrated an upward trend.

This might be due to multitude of reasons. The widespread use of the internet facilitating faster and broader dissemination of knowledge would have certainly contributed to rise in the citation counts of dental journals. The significant rise in the aggregate immediacy index (which indicates how fast articles are being cited) from 0.159 in 2003 to 0.275 in 2013 would further bear witness to effect of internet on citation frequency. In addition, the advances in technology would have facilitated more sophisticated research that might have not been possible in the 1980’s or 1990’s. Also the current ‘publish or perish’ climate in academia where decision on recruitment, promotion, tenure and funding decisions are based on research output would have motivated the dental community’s increased productivity. However, this increased drive to publish has certainly had a positive influence on key bibliometric indices of dental journals; as the number of publications highly correlated with the total number of citations, aggregate IF and aggregate immediacy index.

According to linear regression analysis results the change in the median IF was not significant (p = 0.397) but a significant rise was noted in the aggregate IF. This inconsistency may have resulted due to the differences in the method of calculating these two indices. While the median IF represent only the middle value of all IF's of dental journals in a specific year, the aggregate IF represent the mean value. Since all data points are taken into account for calculating the mean, it can be thrown right out by a few extreme values. When computing the aggregate IF, the number of citations acquired by all dental journals is divided by the number of articles published in the previous two years. Therefore if some top-tier dental journals acquire many citations the aggregate IF will increase, since it is largely influenced by these outliers. According to graph depicting the change in IF’s over the years (Fig. 1d), the median IF of dental journals have grown up to 2007, but declined thereafter. The combined effect of the median IF’s rise and subsequent fall after 2007 would have resulted in its overall changes from 2003 to 2012 being statistically not significant.

The aggregate cited half-life represents the median age of articles cited in a particular JCR year, indicating the turnover rate of the body of work on a subject. The statistically significant decline in the aggregate cited half-life from 2003 to 2012 illustrates that articles published in dental journals have been citing more and more newer studies. This phenomenon may have resulted due to rapid advances in technology which have changed both the clinical practice of dentistry as well as dental research.

The cited half-life of a journal indicates “the median age of the articles that were cited in the JCR year”[7]. For example, if the cited half-life of journal X in 2012 is 8.0, this indicates that 50% of all citations received by this journal in 2012 were from those published in it from 2005 to 2012 (both years inclusive). Likewise aggregate cited half-life aids in evaluating the age of articles cited dental journals. We noted a statistically significant decline in this metric from 2003 to 2012.

The analysis of article types published from 2003 to 2012 revealed some interesting findings. Although a dramatic increase in the number of research articles (β = 422.22) were noted, the number of reviews (β = 39.515) did not grow with the same speed. This would indicate that more and more original research in dentistry has been published through the years compared to reviews, which is an encouraging prospect. On the other hand, reviews attract more citations than research articles as the former is a surrogate of published literature [16]. More citations will inevitably lead to an increase in most of the key bibliometric indices. Thus the question of how editors and publishers of dental journals balance the articles: reviews ratio remain unresolved.

We further analyzed the top-5 and bottom-5 journals in relation to their IF's in 2003. It was interesting to note that most of the top-5 journals retained their percentile rankings with only minor seasonal changes. In contrast, the British Journal of Oral & Maxillofacial Surgery, which was among the ‘underdogs’ in 2003 being ranked 42^nd^, increased its IF significantly throughout the years becoming the 10^th^ highest ranked dental journal in 2012. Except for Clinical Oral Implants Research and Oral Oncology, the number of articles published in the top-5 journals remained relatively constant. Nevertheless, the citations received by all these 10 journals increased significantly from 2003 to 2012. This is in-line with the trend observed for all dental journals.

We did not use a comparison group for this study, even though general medicine or surgery journals would have been a possible choice. As the number of medical or surgical journals that exists and number of citations these receive substantially exceeds the dental specialty, there was no point to perform such a comparison. For example during 2012, those journals under JCR category of “Medicine, General and Internal” had 1,053,562 citations with an aggregate IF of 3.934 while their dental counterparts received 233,232 citations and aggregate IF of 1.739.

Despite its flaws [17] It is impossible to ignore the influence of the IF. The IF has gained recognition as an indicator of journal quality and prestige [18,19]. Although the IF cannot assess the true impact of an individual publication, authors are increasingly submitting their dental related manuscripts to non-dental journals that have high IF’s. A simple search of the Web of Science database with the keywords ‘dental’ or ‘dentistry’ revealed 15,162 papers published in 2012. However, only 7,968 (52.55%) were published in journals belonging to the subject category Dentistry, Oral Surgery and Medicine while the rest (47.45%) were featured on non-dental journals. This results in a “brain drain” as a potential loss to the body of knowledge in dentistry. Research findings published in such non-dental journals may not be readily accessible to dental community unless they specifically searched for a particular topic.

A possible solution to the “Impact Factor Game” [20–23] would be to introduce a standardized IF that could be compared across different disciplines. This could be achieved by dividing each journals IF, by the aggregate IF of the corresponding category. Thus, those journals with a standardized IF greater than 1 could be considered outperforming its peers.

The value of ideas contained in a journal will eventually depend on its usage. Most of the key bibliometric indices are based on citation analysis. However, citations alone are not an accurate reflection of the usage of a particular journal. Individuals may read a journal article; some may even discuss it with colleagues, but may not cite it. Unfortunately, validated indices that track the actual usage of journals/articles among readers are currently unavailable. This may partly be related to the difficulty in accurately determining how many read the print or online version of a paper. Metrics such as online view counts are unreliable as readers may click on an article but may not read it eventually. Furthermore, these click counters are prone to abuse by those who want to artificially inflate their views using automated computer programs [24].

We have presented a number of metrics (other than the IF) for evaluating performance of dental journals. While providing a self-evaluation for the dental community as a whole, the results presented here would be of value to editors and publishers of dental journals. These findings represent the average for the field, which can be used as a comparative benchmark to gauge the success and direction of their respective journals. Such information would be useful when making decisions on editorial policies and practices.

## Conclusion

Significant growth in the dental literature in terms of number of journals, frequency and number of publications and acquired citations was noted. The majority of the key bibliometric indices demonstrated an upward trend. While providing a self-evaluation for the dental community, these findings would be of value to editors and publishers of dental journals as a comparative benchmark for gauging success and direction of their respective journals.
